# Increasing late diagnosis in HIV infection in South Korea: 2000-2007

**DOI:** 10.1186/1471-2458-10-411

**Published:** 2010-07-13

**Authors:** Jin-Hee Lee, Gab Jung Kim, Byeong-Sun Choi, Kee-Jong Hong, Mi-Kyung Heo, Sung Soon Kim, Mee-Kyung Kee

**Affiliations:** 1 Division of AIDS, Korea Centers for Disease Control and Prevention, Seoul, Korea; 2 Division of HIV and TB Control, the Korea Centers for Disease Control and Prevention, Seoul, Korea; 3 Division of Influenza Viruses, Korea Centers for Disease Control and Prevention, Seoul, Korea

## Abstract

**Background:**

The number of Koreans diagnosed with human immunodeficiency virus (HIV) infections is increasing annually; however, CD4+ T-cell counts at diagnosis have decreased. The purpose of the present study was to identify clinical and epidemiologic associations with low CD4+ T-cell counts at the time of HIV diagnosis in a Korean population.

**Methods:**

Data from 2,299 HIV-infected individuals with initial CD4+ T-cell counts measured within 6 months of HIV diagnosis and reason for HIV testing were recorded and measured from 2000 to 2007. Data were selected from the database of the Korea Centers for Disease Control and Prevention. Late diagnosis was defined by CD4+ T-cell counts <200 cells/mm^3^. Reasons for HIV testing were analyzed using logistic regression including epidemiologic variables.

**Results:**

A total of 858 individuals (37.3%) were included in the late diagnosis group. Individuals with a late diagnosis were older, exposed through heterosexual contact, and demonstrated clinical manifestations of acquired immunodeficiency syndrome (AIDS). The primary reason for HIV testing was a routine health check-up (41%) followed by clinical manifestations (31%) of AIDS. The proportion of individuals with a late diagnosis was higher in individuals tested due to clinical symptoms in public health centers (adjusted odds ratio [AOR], 17.3; 95% CI, 1.7-175) and hospitals (AOR, 4.9; 95% CI, 3.4-7.2) compared to general health check-up. Late diagnosis annually increased in individuals diagnosed by voluntary testing both in public health centers (PHCs, P = 0.017) and in hospitals (P = 0.063). Routine testing due to risky behaviors resulted in earlier detection than testing secondary to health check-ups, although this difference was not statistically significant (AOR, 0.7; P = 0.187). Individuals identified as part of hospital health check-ups more frequently had a late diagnosis (P = 0.001)

**Conclusions:**

HIV infection was primarily detected by voluntary testing with identification in PHCs and by testing due to clinical symptoms in hospitals. However, early detection was not influenced by either voluntary testing or general health check-up. It is important to encourage voluntary testing for early detection to decrease the prevalence of HIV infection and AIDS progression.

## Background

Survival in human immunodeficiency virus (HIV)-infected individuals has improved with the introduction of highly active antiretroviral therapy (HAART) [1-3]. However, there is still a high risk of mortality in infected individuals due to late diagnosis. Late diagnosis results in delayed treatment, higher medical costs, and an increased risk of transmission by infected individuals unaware of their infection status [4-7]. A total of 6,120 cumulative HIV infections were identified in Korea as of 2008; 5,036 of these individuals had been previously living with HIV/AIDS. The annual number of newly diagnosed HIV infections has been increasing, while the CD4+ T-cell counts at HIV diagnosis has been steadily decreasing, implying a delayed diagnosis in HIV-infected Koreans [8]. A survival study of HIV-infected Koreans reported that 45% of deaths occurred within 6 months of HIV diagnosis [9], and suggested the presence of delayed diagnosis and antiretroviral therapy in Korea.

The prevalence of HIV in Korea is low compared to many other countries [10-12]; however, the annual number of newly diagnosed individuals has increased from 2000 (n = 219) to 2008 (n = 797). The Korean government has implemented various policies to improve early detection in HIV-infected individuals. Public health centers (PHCs) have exempted patients and individuals in high-risk groups from HIV testing costs and have performed anonymous HIV testing since 1989 [13]. Non-governmental organizations have implemented voluntary counseling and testing programs (VCT) for the general population as well as for high risk individuals [14]. HIV seroconvertors have been identified at screening sites since 2002 through the introduction of viral antigen and antibody detection methods [15]. In addition, the Korean government has strongly enforced HIV prevention policies, including health education and financial support of medical expenses, to HIV-infected individuals [16].

Early diagnosis is required to prevent HIV transmission from infected individuals, and may result in more efficient treatment and improvements in quality of life for infected individuals [9]. The reasons for delayed HIV diagnosis were defined in the present study to improve strategies for earlier diagnosis.

## Methods

### Subjects

Data from PHCs, which perform epidemiological investigations of new HIV diagnosed individuals, were transferred to the Division of HIV & TB Control and registered in the HIV database of the Korea Centers for Disease Control and Prevention (KCDC). In addition, CD4+ T-cell count testing of registered individuals was performed by the Division of AIDS; the data was managed using a KCDC laboratory information management system (LIMS) [16]. A total of 2,299 individuals with initial CD4+ T-cell counts measured within 6 months of diagnosis and a documented reason for testing were identified (from 4,259 HIV-infected individuals registered at the KCDC from 2000 to 2007). CD4+ T-cell counts were used as a marker to estimate the stage of disease progression in the HIV-infected individuals [17-19]. A late diagnosis was defined by a CD4+ T cell count <200 cells/mm^3 ^at the time of diagnosis [9,20,21], according to KCDC guidelines and a survival study of HIV-infected Koreans. Ethics approval was obtained from the KCDC Institutional Review Board (IRB) Ethics Committee.

### Statistical Analyses

Epidemiological variables associated with late HIV diagnosis were analyzed, including gender, age at HIV diagnosis, transmission route, testing region, screening site, and reason for testing. Screening sites were categorized into three groups: PHCs, hospitals, and blood centers. Transmission routes were classified into three groups: heterosexual, homosexual (including bisexual), and blood transfusions (or use of blood products). Reasons for HIV testing were classified into fifteen groups based on KCDC guidelines for HIV/AIDS control [16], and groups were separated into five categories (Table 1).

**Table 1:** Classification of the reasons for HIV testing in Korea (2000-2007)

Category	Reason for HIV testing
Health check-ups	**- General health check-up**: A comprehensive physical examinations
	**- Medical certificate: **Medical certificates for workplace permits, residence halls, licenses, officers, workers, welfare centers, detention centers, and shelters for women.
	**- Prenatal check-up**
	**- Prisoner status**
	**- Operations: **Testing prior to surgical procedures in hospitals
	**- Blood donation**^**a **^**: **Routine testing blood for transfusion at blood centers

Knowledge of status	**- Voluntary testing with ID: **Testing with identification (ID) because individuals wanted to know their HIV status.
	**- Anonymous: **Testing anonymously because they wanted to know their HIV status at public health center (PHC), but registered in Korea Centers for Disease Control and Prevention after diagnosis to get governmental support of medical care.

Risky behaviors	**- STI risk groups: **Commercial sex workers, bar employees, tea-room employees, massage parlor employees tested routinely at PHC
	**- STD patients: **Additional HIV testing while undergo STD treatment at PHC
	**- HIV-infected partners: **Testing due to partner of HIV-infected individual at PHC

Clinical manifestation	**- Symptoms: **Testing due to clinical symptoms
	**- Physician's referral: **Referral by physician due to clinical suspicion of HIV
	**- Tuberculosis: **Testing for tuberculosis patients

STI: Sexually transmitted infection, STD: Sexually transmitted diseases.

^a^Blood donation: Blood check-up for transfusion

Trend tests were used to evaluate the linearity for the proportion of late diagnoses in hospitals and PHCs. We analyzed the association between late diagnosis and epidemiological variables using multiple logistic regressions to rule out confounding effects. Also, late diagnosis was analyzed by reason for HIV testing, and the general health check-up group was used as a reference group (tested during comprehensive physical examinations). The two-tailed test was used to identify statistical significance with a 95% confidence interval, and the SAS software (version 9.1) was used for all statistical analyses.

## Results

### Epidemiological characteristics of study population and late diagnosis

The primary characteristics of the study population had a similar distribution to the general population of HIV-infected individuals from 2000-2007 (Table 2). The majority of infected individuals were men (92%). The highest proportion of infected individuals were 30-39 years old (32%), metropolitan dwellers (69%), and underwent HIV testing in hospitals (66%). The primary reasons for HIV testing were health check-ups (41%) and presence of clinical manifestations (31%). The late diagnosis group included 858 individuals (37.3% of the study population). Older groups had a higher proportion of late diagnosis: the adjusted odds ratio (AOR) was 1.7 for the 30-39-year-old group (P < 0.001), 2.6 for the 40-49-year-old group (P < 0.001), and 2.7 for ≥50-year-old group (P < 0.001). The 15-29-year-old group was used as a reference group. Individuals infected by homosexual contact were less likely to have a low CD4+ T-cell count (<200 cells/mm^3^) at diagnosis than those infected by heterosexual contact (AOR, 0.8; P = 0.045).

**Table 2:** Epidemiological characteristics of late diagnosis in HIV-infected individuals in Korea (2000-2007)

Characteristic	Total No. (%)	Study population No. (%)	Late diagnosis		Unadjusted		Adjusted	
			
			No.	%*	OR (95% CI)	P-value	OR(95%CI)	P-value
Total	4259	2299	858	37.3	-	-	-	-

Gender								
Male	3935 (92)	2108 (92)	798	37.9	Reference	-	Reference	-
Female	324 (8)	191 (8)	60	31.4	0.8 (0.5-1.0)	0.079	0.8 (0.6-1.2)	0.291

Age at HIV diagnosis								
15-29	984 (23)	617 (27)	128	20.8	Reference	-	Reference	-
30-39	1386 (33)	734 (32)	248	33.8	2.0 (1.5-2.5)	<.001	1.7 (1.3-2.3)	<.001
40-49	1025 (24)	525 (23)	258	49.1	3.7 (2.8-4.8)	<.001	2.6 (1.9-3.5)	<.001
50≤	864 (20)	423 (18)	224	53	4.3 (3.3-5.6)	<.001	2.7 (2.0-3.7)	<.001

Transmission route^§^								
Heterosexual	2259 (59)	1189 (56)	412	34.7	Reference	-	Reference	-
Homosexual	1585 (41)	917 (44)	268	29.2	0.8 (0.6-0.9)	0.008	0.8 (0.7-0.9)	0.045
Other^†^	13 (0)	5 (0)	2	40	1.3 (0.2-7.6)	0.802	2.1 (0.3-14)	0.442

Region								
Metropolitan	3021 (71)	1595 (69)	593	37.2	Reference	-	Reference	-
Small town or rural	1238 (29)	704 (31)	265	37.6	1.0 (0.8-1.2)	0.832	0.8 (0.7-1.0)	0.117

Screening site								
Public health center	868 (20)	545 (24)	154	28.3	Reference	-	Reference	-
Hospital	3047 (72)	1529 (66)	676	44.2	2.0 (1.6-2.5)	<.001	0.9 (0.7-1.3)	0.657
Blood center	344 (8)	225 (10)	28	12.4	0.4 (0.2-0.6)	<.001	0.5 (0.3-0.8)	0.003

Reason for testing								
Health check-ups	1466 (40)	940 (41)	231	24.6	Reference	-	Reference	-
Knowledge of status^a^	561 (15)	377 (16)	114	30.2	1.3 (1.0-1.7)	0.035	1.1 (0.8-1.6)	0.421
Risky behaviors^b^	276 (8)	187 (8)	38	20.3	0.8 (0.5-1.2)	0.213	0.7 (0.4-1.1)	0.187
Clinical manifestation^c^	1104 (30)	700 (31)	450	64.3	5.5 (4.4-6.8)	<.001	3.0 (2.4-3.8)	<.001
Others	259 (7)	95 (4)	25	26.3	1.1 (0.7-1.8)	0.708	0.9 (0.6-1.6)	0.827

Late diagnosis was CD4+ T cell count <200 cells/mm^3 ^at the time of HIV diagnosis.

* % was the proportion of HIV-infected individuals with late diagnosis divided by total cases.

^§ ^Data without the information of transmission route was not presented.

^† ^Other at transmission route: HIV-infected individuals by transfusion and blood products.

OR: Odds ratio, CI: Confidence Interval.

In the number of total, the proportions for "Transmission route" and "Reason for testing" were calculated without missing cases (402, 593 cases).

In the number of study population, the proportions of transmission route were calculated without missing cases (188 cases were not surveyed).

^a^Knowledge of status: Testing because individuals wanted to know their HIV status.

^b^Risky behaviors for HIV infection.

^c^Clinical manifestation of HIV infection.

Individuals tested due to clinical manifestations were diagnosed later than individuals tested during routine health check-ups (AOR, 3.0; P < 0.001). Testing due to risky behaviors resulted in earlier detection of infection than testing during health check-ups, although the difference was not statistically significant (AOR, 0.7; P = 0.187). There was no difference between individuals tested for knowledge of status and individuals tested as part of routine health check-ups (AOR, 1.1; P = 0.421). There were significant differences in the proportion of individuals with a late diagnosis as analyzed by screening site (OR, 2.0; P < 0.001 in hospital, or OR, 0.4; P < 0.001 in blood centers), although differences were no longer statistically significant in the hospital upon adjustment for confounders (AOR, 0.9; P = 0.657).

### Late diagnosis and reason for HIV testing

**Figure 1:**
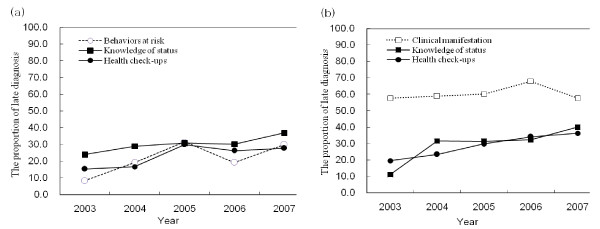
**Trend for the proportion of late diagnosis of HIV-infected individuals stratified by reason for HIV testing (2003-2007)**. (a) Public Health Centers, including health check-ups (general health check-up, medical certificate, prenatal check-up, prisoner status, P= 0.096), knowledge of status (voluntary test with identification, anonymous testing*, P = 0.017) and risky behaviors* (STI risk groups, STD patients, and HIV-infected partners, P = 0.166). (b) Hospital, including health check-ups (general health check-up, medical certificate, prenatal check-up, medical operation, P = 0.001), knowledge of status (voluntary test with identification, P = 0.063), and clinical manifestation of HIV infection (symptom, physician's referral, tuberculosis, P = 0.584). *Risky behaviors (STI risk groups, STD patients, and HIV-infected partners), prisoner status, and anonymous HIV testing performed at PHCs. Late diagnosis was defined by CD4+ T cell counts <200 cells/mm^3 ^at the time of HIV diagnosis. Knowledge of status: Testing performed because individuals wanted to know their HIV status. A trend identifying the clinical manifestations of HIV infection in PHCs was not identified. This may have been due to the small number of cases (n = 30).

We analyzed late diagnosis by several characteristics, including reason for HIV testing in PHC and hospital. The median CD4+ T-cell counts of individuals at the time of HIV diagnosis was 252 cells/mm^3 ^(IQR; 102-407). The proportion of individuals assigned a late diagnosis who were diagnosed in PHCs (28.3%) was significantly lower than the proportion of individuals diagnosed in hospitals (44.2%) (P = 0.001) (Table 3). In PHC, Individuals who underwent voluntary testing with identification (29.5%) comprised the largest group of diagnosed individuals; the proportion of individuals with a late diagnosis was not different from individuals diagnosed as part of a general health check-up (AOR, 1.1; 95% CI: 0.5-2.5). However, among individuals diagnosed due to clinical symptoms the proportion of late diagnosis was highest in PHCs (90.9%, AOR, 17.3; 95% CI: 1.7-175). A large proportion of individuals diagnosed in hospitals had clinical symptoms (26.9%), and the proportion of individuals with a late diagnosis was higher in individuals tested due to tuberculosis (AOR, 6.1; 95% CI: 1.1-35.7), clinical symptoms (AOR, 4.9; 95% CI: 3.4-7.2), or physician's referral (AOR, 1.7; 95% CI: 1.2-2.4) compared to those diagnosed as part of general health check-up. There were no significant differences in late diagnosis stratified by screening site (data not shown). The annual trend of late diagnosis based on reason for testing at each screening site is displayed in Figure 1. Late diagnosis steadily increased in individuals tested for knowledge of their HIV status (Figure 1A, black square with solid line; P = 0.017) in PHCs. In addition, late diagnosis in individuals tested for knowledge of their HIV status in hospital showed an increasing trend which was not statistically significant (Figure 1B, black square with solid line; P = 0.063). Late diagnosis was increasing in those detected during the health check-ups in the hospital (Figure 1B, black circle with solid line; P = 0.001).

**Table 3:** Characteristics of late diagnosis by reason for HIV testing of individuals diagnosed in public health center and hospital in Korea (2000-2007)

Category	Reason for HIV testing	Total (N = 2,074)		**AOR**^**a**^ (95%CI)	Public health center (N = 545)		Hospital (N = 1,529)	
		Median CD4+ count (IQR)	No. (Late diagnosis; %)		No. (Late diagnosis; %)	**AOR**^**a**^ (95%CI)	No. (Late diagnosis; %)	**AOR**^**a**^ (95%CI)
Total		252 (102-407)	2,074 (40.0)	-	545 (28.3)	-	1,529 (44.2)	-
Health check-ups	General health check-up	280 (174-428)	325 (29.2)	Reference	36 (30.6)	Reference	289 (29.1)	Reference
	Medical certificate	304 (196-460)	138 (26.1)	1.0 (0.6-1.6)	36 (25.0)	0.8 (0.3-2.4)	102 (26.5)	1.0 (0.6-1.8)
	Prenatal check-up	482 (428-649)	9 (11.1)	0.4 (0.1-4.1)	1 (0)	-	8 (12.5)	0.4 (0.0-4.1)
	Prisoner status	383 (298-500)	25 (16.0)	0.3 (0.1-0.9)	25 (16.0)	0.3 (0.1-1.4)	-	-
	Operation	297 (176-443)	218 (30.7)	1.1 (0.8-1.7)	-	-	218 (30.7)	1.1 (0.8-1.7)
Knowledge of stauts^b^	Voluntary test with ID	308 (166-442)	314 (30.9)	1.1 (0.8-1.6)	161 (32.9)	1.1 (0.5-2.5)	153 (28.8)	1.1 (0.7-1.8)
	Anonymous	258 (186-375)	63 (27.0)	0.9 (0.5-1.9)	63 (27.0)	0.9 (0.4-2.5)	-	-
	STI risk groups	416 (286-569)	60 (11.7)	0.4 (0.2-0.9)	60 (11.7)	0.5 (0.2-1.7)	-	-
Risky behaviors^c^	STD patients	308 (198-461)	75 (25.3)	0.8 (0.4-1.6)	75 (25.3)	0.8 (0.3-2.1)	-	-
	HIV-infected partner	370 (214-566)	52 (23.1)	0.6 (0.2-1.3)	52 (23.1)	0.8 (0.2-2.2)	-	-
	Symptoms	127 (38-160)	422 (78.7)	5.1 (3.6-7.4)	11 (90.9)	17.3 (1.7-175)	411 (78.3)	4.9 (3.4-7.2)
Clinical manifestations^d^	Physician's referral	226 (104-354)	263 (41.1)	1.7 (1.2-2.4)	10 (40.0)	1.8 (0.4-8.5)	253 (41.1)	1.7 (1.2-2.4)
	Tuberculosis	151 (48-326)	15 (66.7)	5.5 (1.7-18)	9 (66.7)	5.4 (1.0-30.1)	6 (66.7)	6.1 (1.1-35.7)
Others	Others	295 (148-437)	95 (26.3)	1.0 (0.6-1.7)	6 (33.3)	295 (149-433)	89 (25.8)	1.0 (0.5-1.7)

Late diagnosis was CD4+ T cell count <200 cells/mm^3 ^at the time of HIV diagnosis.

Variables in the model: sex, age at diagnosis, transmission route, testing region, reason for testing.

* % was proportion of HIV-infected individuals with late diagnosis divided by total cases.

ID: Identification, STI: Sexually transmitted infection, STD: Sexually transmitted diseases.

IQR: Interquantile range, CI: Confidence Interval.

^a^Adjusted odds ratio(AOR): Test result for proportion of late diagnosis in total HIV infection.

^b^Knowledge of status: Testing because individuals wanted to know their HIV status.

^c^Risky behaviors for HIV infection.

^d^Clinical manifestation of HIV infection.

## Discussion

HIV prevalence in developed countries has been increasing due to continuous detection of newly diagnosed individuals and prolonged survival of HIV-infected patients. This increased lifespan has caused an increased burden on health care in many countries, including Korea [22-24]. Accordingly, the Korean government has spent additional funds on treatment, and the current estimated lifetime cost per HIV-infected individual is approximately 0.4 million US dollars [24]. The late initiation of treatment of HIV-infected individuals due to late diagnosis may accelerate other chronic disorders and infections [25-27], although HAART decreases the frequency of AIDS-related opportunistic infection. This burden may be lessened through earlier detection of HIV infection. We attempted to clarify reasons for late diagnosis in a large group of Korean individuals in the present study.

A greater proportion of individuals with a late diagnosis were identified in older and heterosexual individuals as well as individuals who were tested due to AIDS symptoms. Older individuals were likely to have sustained infection at younger ages, but had a delayed diagnosis. Individuals who underwent routine HIV testing had a low risk of late diagnosis. HIV detection in young women at risk for STIs and young male blood donors was earlier than others due to mandatory testing [28], and regular physical examinations of pregnant women were another reason for early HIV diagnosis. The risk of late diagnosis was significantly affected by transmission route; homosexual contacts had a greater proportion of early detection, likely secondary to frequent testing [29]. Previous studies have reported a high proportion of delayed diagnosis in patients with AIDS symptoms, physician's referrals, and clinical tuberculosis, similar to our results [18,20,28].

The reasons for HIV testing differed between PHCs and hospitals. PHCs handled testing in the local community based on national HIV prevention policies; HIV tests were also provided to individuals at risk of STIs, prisoners, tuberculosis patients, low-income individuals, and residents in welfare shelters in PHCs. Individuals could also be tested anonymously in PHCs.

We analyzed the characteristics of individuals by diagnosis in PHCs or in hospitals. Routine testing contributed to the early diagnosis of HIV in the STI risk group, STD patients, partner of HIV-infected individuals, prisoners, and prenatal women in PHCs. Therefore, an improved national policy will enhance the early diagnosis of HIV infection. Approximately five million (70%) HIV tests are performed annually in hospitals as part of a health check-up and as part of medical procedures [30]; however, many individuals were only diagnosed during the late stages of HIV infection. The proportion of late diagnoses in these individuals appeared to be increasing.

We previously reported that the initial CD4+ T-cell counts of HIV-infected individuals at diagnosis have decreased annually [4]. Our results demonstrated that 37% of individuals in the present study had a CD4+ T-cell count <200cells/mm^3 ^at diagnosis, while 16% had a CD4+ T cell count <50 cells/mm^3 ^at diagnosis. Approximately 42% of individuals with HIV infections had clinical symptoms of AIDS at diagnosis or within 1 year after diagnosis [19] in a U.S. of intravenous drug users, and the proportion of delayed diagnosis was elevated in Switzerland, Canada and France [5,21,31]. Approximately 25% of HIV-infected individuals in the U.S. were unaware of their infection status, and the transmission rate was 3.5 times higher in this unaware group [7]. The prolonged period from infection to diagnosis increases the transmission rate to other individuals, while most individuals aware of a positive HIV status may alter risky sexual behaviors [6,7,32]. This suggests that early detection contributes to the reduction of HIV transmission. HIV testing status, social and personal knowledge of HIV/AIDS, the rights of HIV-infected individuals, and effective health and medical systems may collectively contribute to the reduction of HIV transmission.

This study had two limitations. First, we defined late diagnosis as case of CD4+ T cell count <200/cells/mm^3 ^at the time of HIV diagnosis. Although many studies have used this definition of delayed diagnosis [9,20,21], this may not be completely accurate. Additionally, it is likely that there were some bias in the classification of the transmission route among HIV-infected individuals. Homosexual transmission made up 44% in the present study, and this may be an underestimated value [33]. The primary route of HIV transmission was through sexual contact (99%), and the proportion of HIV-infected men was 91% in Korea. Therefore, homosexual transmission appears to be the primary mode of sexual transmission of HIV infection in Korea.

## Conclusions

It is important for physicians and other public health professionals to emphasize HIV testing in older individuals. Physicians and medical professionals must emphasize awareness of symptom-related HIV diagnosis since the majority of testing in Korea takes place in hospitals. To encourage the uptake of HIV testing, several suggestions have to be considered. Health insurance institute must subsidize hospital-based HIV testing for general health check-up. Secondly, the testing consent process should be simplified for HIV testing, similar to processes for other hospital-administered tests. Lastly, extensive education and campaigns to expand voluntary testing will enhance earlier testing.

## Competing interests

The authors declare that they have no competing interests.

## Authors' contributions

MKK and JHL designed and conceived the idea for the study and MKK supervised all aspects of its implementation. JHL completed the all data analyses and wrote the first draft of the manuscript. MKK and SSK coordinated funding for the project. SSK contributed to the critical revising for important intellectual content. GJK and BSC were responsible for the immune data acquisition and contributed to the interpretation of the collected data. KJH also contributed to the critical revising for important intellectual content and discussion of disease progression. MKH contributed to the interpretation and collection of early (raw) data. All authors edited and approved the final version of the manuscript.

## Pre-publication history

The pre-publication history for this paper can be accessed here:

http://www.biomedcentral.com/1471-2458/10/411/prepub
